# Pharmacogenetically Guided Escitalopram Treatment for Pediatric Anxiety Disorders: Protocol for a Double-Blind Randomized Trial

**DOI:** 10.3390/jpm11111188

**Published:** 2021-11-12

**Authors:** Jeffrey R. Strawn, Ethan A. Poweleit, Jeffrey A. Mills, Heidi K. Schroeder, Zoe A. Neptune, Ashley M. Specht, Jenni E. Farrow, Xue Zhang, Lisa J. Martin, Laura B. Ramsey

**Affiliations:** 1 Anxiety Disorders Research Program, Department of Psychiatry & Behavioral Neuroscience, College of Medicine, University of Cincinnati, Cincinnati, OH 45219, USA; strawnjr@uc.edu (J.R.S.); heysehk@uc.edu (H.K.S.); neptunza@uc.edu (Z.A.N.); huckabam@uc.edu (A.M.S.); farrowje@uc.edu (J.E.F.); 2 Cincinnati Children’s Hospital Medical Center, Department of Pediatrics, Division of Clinical Pharmacology, Cincinnati, OH 45219, USA; poweleen@mail.uc.edu; 3 Cincinnati Children’s Hospital Medical Center, Department of Pediatrics, Division of Child & Adolescent Psychiatry, Cincinnati, OH 45219, USA; 4 Cincinnati Children’s Hospital Medical Center, Department of Pediatrics, Division of Biomedical Informatics, College of Medicine, University of Cincinnati, Cincinnati, OH 45219, USA; 5 Department of Biomedical Informatics, College of Medicine, University of Cincinnati, Cincinnati, OH 45219, USA; 6 Department of Economics, Lindner College of Business, University of Cincinnati, Cincinnati, OH 45219, USA; millsjf@uc.edu; 7 Cincinnati Children’s Hospital Medical Center, Division of Human Genetics, College of Medicine, University of Cincinnati, Cincinnati, OH 45219, USA; xue.zhang@cchmc.org (X.Z.); lisa.martin@cchmc.org (L.J.M.); 8 Cincinnati Children’s Hospital Medical Center, Division of Research in Patient Services, College of Medicine, University of Cincinnati, Cincinnati, OH 45219, USA

**Keywords:** selective serotonin reuptake inhibitor (SSRI), anxiety disorders, generalized anxiety disorder (GAD), pharmacogenetic, CYP2C19, pharmacokinetic, tolerability, side effects

## Abstract

Current pharmacologic treatments for pediatric anxiety disorders (e.g., selective serotonin reuptake inhibitors (SSRIs)) frequently use “one size fits all” dosing strategies based on average responses in clinical trials. However, for some SSRIs, including escitalopram, variation in CYP2C19 activity produces substantial variation in medication exposure (i.e., blood medication concentrations). This raises an important question: would refining current SSRI dosing strategies based on CYP2C19 phenotypes increase response and reduce side effect burden? To answer this question, we designed a randomized, double-blind trial of adolescents 12–17 years of age with generalized, separation, and/or social anxiety disorders (N = 132). Patients are randomized (1:1) to standard escitalopram dosing or dosing based on validated CYP2C19 phenotypes for escitalopram metabolism. Using this approach, we will determine whether pharmacogenetically-guided treatment—compared to standard dosing—produces faster and greater reduction in anxiety symptoms (i.e., response) and improves tolerability (e.g., decreased risk of treatment-related activation and weight gain). Secondarily, we will examine pharmacodynamic variants associated with treatment outcomes, thus enhancing clinicians’ ability to predict response and tolerability. Ultimately, developing a strategy to optimize dosing for individual patients could accelerate response while decreasing side effects—an immediate benefit to patients and their families. ClinicalTrials.gov Identifier: NCT04623099.

## 1. Introduction

Anxiety disorders are the most common mental health conditions in youth, affecting up to 15% of children and adolescents [1,2,3] and, when untreated, result in persistent disability [4,5]. Early treatment of pediatric anxiety disorders has the potential to offset the substantial negative effects on the health and wellbeing of children and families. Currently, both cognitive behavioral therapy and antidepressant medications (e.g., selective serotonin reuptake inhibitors (SSRIs) and selective serotonin norepinephrine reuptake inhibitors (SNRIs)) present the most evidence for treating anxiety disorders in youth [6,7,8].

While all antidepressants decrease anxiety in youth, compared to SNRIs, SSRIs produce larger and earlier improvement in anxiety symptoms [9]. This is consistent with treatment recommendations that SSRIs are the first-line psychopharmacologic interventions in pediatric anxiety disorders. However, in prospective, randomized trials of SSRIs [10] as well as in our retrospective study of the SSRI escitalopram in pediatric anxiety disorders [11], only 50–60% of patients respond to these medications. Considering the relationship between SSRI exposure and response in pediatric anxiety and depressive disorders [12], pharmacogenetic variants in drug metabolizing enzymes—that determine exposure (i.e., plasma concentrations)—may help to predict treatment response.

Escitalopram is metabolized by several enzymes in the cytochrome P450 (CYP) system, with CYP2C19 and CYP3A4 contributing the most to the formation of inactive metabolites [13,14]. In patients with reduced CYP2C19 metabolism, escitalopram plasma concentrations are higher than in patients with normal CYP2C19 metabolism, putting these patients at higher risk of SSRI-related side effects. Additionally, there are patients with faster than normal metabolism and lower plasma escitalopram concentrations who are at high risk of treatment failure [15]. In adults, the influence of CYP2C19 metabolizer status on escitalopram pharmacokinetics (PK) is well-described [16,17]; however, there is less evidence on whether these variants are associated with treatment efficacy, especially in youth [18,19]. Moreover, in our prospective study of escitalopram in adolescents with anxiety, intermediate metabolizers improved by 10% more than normal metabolizers given the same dosing regimen [20].

Due to the frequency of SSRI-related side effects [21], clinicians often initiate SSRIs at low doses and slowly titrate these medications until either encountering a side effect or a response. If intolerable side effects occur, the SSRI dose is decreased, or the medication is discontinued. Slow titration minimizes side effects but risks under-treatment in many patients at the start of treatment and the lack of perceived treatment response may ultimately lead to a medication change, further delaying response [22]. Side effects are associated with SSRI concentrations (including escitalopram) in children and adolescents [12,23], as well as adults [24,25,26,27].

In pediatric patients, SSRI-related side effects include weight gain and activation [23,28,29,30]. The latter represents a hyperarousal event that is characterized by hyperactivity, impulsivity, disinhibition, restlessness, and/or insomnia [23,29]. Activation, which is markedly more common in pediatric patients than in studies of adults [29], impairs psychosocial functioning and decreases the likelihood of medication response [31]. SSRI-related activation emerges early in treatment (typically within the first 4–6 weeks) [23,29] or following a dose increase [23], and symptoms may attenuate with dose reduction and/or resolve with discontinuation of the SSRI [29,32]. This temporal pattern is consistent with one prospective study in which higher plasma concentrations of the SSRI, fluvoxamine, were associated with activation [23]. Additionally, Sakolsky et al. found that higher venlafaxine plasma concentrations were associated with specific side effects in adolescents [12]. In our study of prospectively treated adolescents with GAD (*n* = 18) patients who experienced activation had higher escitalopram C_MAX_ and AUC_0–24_ [20]. However, monitoring plasma SSRI concentrations is rarely seen in clinical practice. Thus, identifying factors that increase plasma concentrations (e.g., pharmacogenetic factors) could decrease the risk of these side effects. In adolescents, es/citalopram is significantly associated with increased BMI and greater weight gain compared to other SSRIs [33]. However, escitalopram-related weight gain varies [34] and may be obscured by symptoms of the underlying disorder (e.g., decreased appetite) or the short duration of most pediatric trials [35,36]. For example, in a patient with decreased appetite and weight loss (associated with core symptoms of major depressive disorder), weight gain could be confounded or obscured by improvement in neurovegetative symptoms.

The extant data supporting genotype-outcome associations are generated in adults [37,38] because psychiatric pharmacogenetic studies rarely include pediatric patients. Multiple studies in adults have analyzed the association between pharmacokinetic (e.g., genes affecting plasma concentrations, CYP2D6 and CYP2C19) and pharmacodynamic genes with outcomes (e.g., genes affecting the serotonin pathway, *SLC6A4* and *HTR2A*). The most commonly investigated pharmacodynamic gene is *SLC6A4*, encoding a serotonin transporter that is the target of SSRIs; furthermore, the expression level of the *SLC6A4* gene influences response to SSRIs [39,40,41].

**Figure 1:**
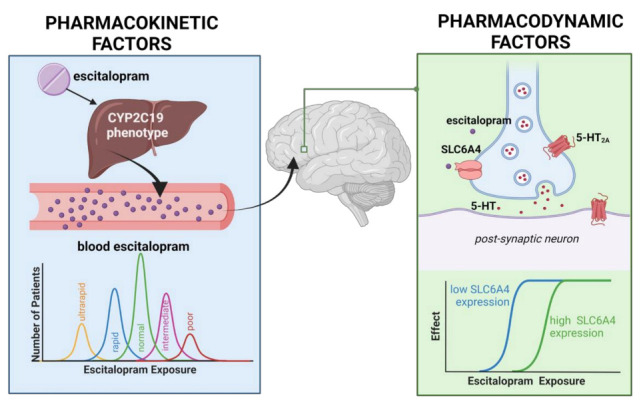
Multiple factors influence the efficacy and side effect profile of escitalopram in anxious adolescents. 5-HT, serotonin; *SLC6A4*, serotonin transporter. The current study primarily focuses on pharmacokinetic factors that impact response and tolerability (blue box), although, secondarily, pharmacodynamic influences (green box) are examined. Image created using Biorender.com.

When the expression of the drug target is low, less SSRI is necessary to produce a response, and when the expression is high, a higher SSRI concentration is necessary to inhibit the target and to produce the desired effect (Figure 1). A 44-base pair insertion in the promoter of *SLC6A4* influences its expression, where the longer allele (with the insertion) is associated with higher expression, better response, and fewer side effects [42]. Additionally, there is a single nucleotide polymorphism (SNP), rs25531, that influences the expression of the longer allele [43]. Together, there are three alleles for *SLC6A4* termed L_A_, L_G_, and S. Variants in the *HTR2A* gene, encoding the serotonin receptor 2A (5-HT_2A_), have inconsistently been associated with es/citalopram response [39,44,45,46,47].

The critical question facing clinicians is: would refining current antidepressant dosing strategies increase treatment response and reduce side effect burden? We propose that a substantial barrier to treating anxiety disorders with SSRIs is that current treatment approaches employ a “one size fits all” dosing strategy based on average responses in clinical trials. However, the identification of pharmacogenetic phenotypes related to response and side effect risks (as well as increased or decreased escitalopram exposure) allows clinicians to assess tolerability and optimize dosing for the individual patient. Ultimately, such a strategy may accelerate response while decreasing side effects—an immediate benefit to patients and their families. Using a randomized, double-blind clinical trial with dosing based on validated phenotypes for escitalopram metabolism, our study facilitates personalized dosing compared. This contrasts with current approach: “standard’’ initial escitalopram dose followed by dose titration until either encountering a response or treatment-limiting side effects. Using this approach, we may also identify pharmacodynamic variants associated with treatment outcomes, thus enhancing our ability to predict response and tolerability. Building pharmacokinetic models in pediatric patients that consider CYP2C19 metabolizer status will facilitate optimal dosing regimens and could be used to predict individual exposure in the future. Finally, this study has the potential to provide a critical foundation of knowledge that will benefit clinical practice and enhance antidepressant treatment response, safety, and tolerability in youth with other disorders (e.g., major depressive disorder).

To assess the impact of pharmacogenetically-guided dosing on adolescents with anxiety disorders, we designed a randomized, controlled trial that compares pharmacogenetically-guided escitalopram dosing to standard dosing with regard to efficacy, tolerability, and safety and explore pharmacodynamic associations with responses. This approach is unique in that there have been no similarly designed trials in adults that address outcomes associated with CYP2C19-guided dosing of escitalopram. While adults and adolescents with anxiety share clinical features and treatment strategies, the lifetime burden in years of poor treatment outcomes is substantially greater in the adolescent population. The demonstration of clinical utility of pharmacogenetically-guided dosing is necessary for widespread adoption and insurance coverage of pharmacogenetic testing [48]. This trial has the potential to provide evidence needed for widespread adoption of pharmacogenetic testing in pediatric patients, increasing the likelihood that adolescents will respond to escitalopram (one of the most commonly prescribed SSRIs in youth) [49,50].

## 2. Study Protocol

### 2.1. Setting and Study Population

The study was conducted at a single, outpatient site and recruited adolescents (age 12–17 years, inclusive) with generalized, separation, and/or social anxiety disorder (pediatric anxiety triad) [51]. Exclusion criteria included patients with >1 prior SSRI trials of adequate dose and duration; a clinically significant, prolonged QTc; co-occurring *DSM-5* mood, eating, bipolar, or psychotic disorder; intellectual disability; history of alcohol or substance use disorder within six months of screening (nicotine use is permitted); female patients of childbearing potential who were sexually active and not practicing a reliable method of contraception; patients who were pregnant, breast feeding, or lactating; or patients who were unable to attend the study visits.

The patients included in the trial met *DSM-5* criteria for generalized, separation, and/or social anxiety disorder diagnosed by the *MINI-KID* [52] with no lifetime history of mania, obsessive compulsive disorder (OCD), or significant history of trauma. Additionally, the patients had a baseline Pediatric Anxiety Rating Scale (PARS) score ≥15 and had not begun psychotherapy within eight weeks of screening. Written, informed consent and assent were obtained from all patients and their legal guardian(s). A buccal swab of the patient’s cheek was collected and sent to a CAP/CLIA-certified facility for genotyping for 7 CYP2C19 no-function alleles (*2, *3, *4, *5, *6, *7, and *8) and the increased function allele (*17), with a *1 genotype inferred from the absence of the previous alleles. The genotype-to-phenotype translation was performed according to the Clinical Pharmacogenetics Implementation Consortium standard [53]. The first patient was enrolled on 8 March 2021, and enrollment is expected to be completed in June 2025.

### 2.2. Randomization

When considering the optimal randomization strategy, we balanced factors that may influence the outcomes while ensuring that the most important aspects were accounted for in the initial randomization. As metabolizer status affects the dose a patient receives, it is critical to account for metabolizer status during randomization. Prior studies have also demonstrated that response to treatment may differ by sex [54]. While we recognize the importance of age and race as other potential factors, the literature on these effects is more limited; thus, they were not considered in the randomization. Patients were randomized (1:1) to a standard or a pharmacogenetically-guided escitalopram treatment.

### 2.3. Medication and Dosing

We selected escitalopram, a Food and Drug Administration (FDA) approved SSRI in adolescents [36] because it is commonly prescribed [49] and it has been extensively studied in pediatric patients with anxiety disorders/major depressive disorder, and pediatric pharmacokinetic data are available [55].

**Figure 2:**
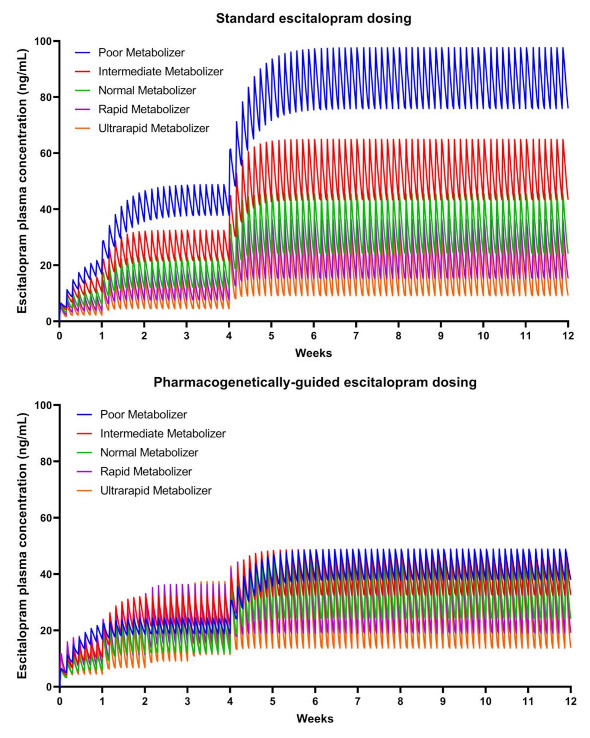
Standard (**top**) and pharmacogenetically-guided dosing (**bottom**) in adolescents result in considerable variation and harmonization of the concentration–time curves, respectively. Adapted from Strawn et al., 2017.

The treatment was double-blind, and escitalopram tablets were over-encapsulated to maintain the blind. The pharmacogenetically-guided dosing was based on published pharmacokinetic models [56] that incorporate CYP2C19 activity as well as age in adolescents [57,58]. These pharmacokinetic models standardize escitalopram exposure across CYP2C19 phenotypes, with normal metabolizers receiving 20 mg/day as the reference. Exposure, at steady state, varies by ≤8% between each phenotype and that of a normal metabolizer (Figure 2).

In patients randomized to standard dosing, escitalopram was prescribed, consistent with the dosing strategy used in the adolescent registration trial that gave rise to the FDA approval for escitalopram in this age group [36]. In patients randomized to pharmacogenetically-guided escitalopram dosing, titration was based on the patient’s CYP2C19 phenotype and predicted escitalopram exposure [56] (Figure 2).

### 2.4. Primary Outcome

Efficacy was assessed with both continuous (i.e., PARS) [59] and categorical measures of response (i.e., Clinical Global Impression-Improvement (CGI-I) and severity (CGI-S)) [60]. The CGI-I and CGI-S [60] were chosen because they have been used in most federally and industry-funded trials of pediatric anxiety disorders [10,61,62,63,64] and are reliably associated with decreases in anxiety and functional recovery in pediatric patients with the disorders being targeted in this study [65,66]. Thus, we chose an endpoint that is clear, informative, and relevant to clinical care and our hypothesis.

### 2.5. Secondary Outcomes

Activation was measured, at each visit, with the Treatment-Emergent Activation and Suicidality Assessment Profile (TEASAP) [67]. BMI was determined over a 12-week period and was used rather than the BMI percentile or the BMI z-score as we do not expect age-related changes in weight during the 12 weeks of the study. Vital signs, co-occurring depressive symptoms, and any worsening of anxiety were assessed at each visit.

Suicidality was monitored at every visit with the Columbia Suicide Severity Rating Scale (CSSRS) [68], consistent with prior trials of youth with anxiety and depressive disorders [20,63,69]. Additionally, while meta-analyses have consistently failed to identify an increased risk of suicidality in SSRI-treated adolescents with anxiety disorders [70,71], all SSRIs currently have FDA “boxed warning” related to “suicidal thinking and behavior.” Recent data suggest that—when present—the possible risk of suicidality differs among antidepressant medications [71], with the highest rates being observed for paroxetine and venlafaxine. This was an additional factor that influenced our choice to use escitalopram.

We determined how *HTR2A* and *SLC6A4* alleles that influence serotonin signaling predict response, when controlling for escitalopram exposure at a given dose. We expect that patients with alleles associated with low *SLC6A4* and high *HTR2A* expression respond well with a lower exposure of escitalopram compared to patients with high *SLC6A4* and low *HTR2A* expression, as we saw in our prospectively treated cohort [20]. We also expect that patients with low *SLC6A4* expression and high *HTR2A* expression alleles experience more side effects (activation and more weight gain) than patients with high *SLC6A4* expression and low *HTR2A* expression when exposure to escitalopram is high, based on off-target effects observed in paroxetine-treated adults [41].

### 2.6. Hypotheses and Statistical Analyses

The change in anxiety severity over the study was evaluated with mixed models. To evaluate differences between treatment arms, we included a time by study arm interaction in the model as well as a study arm.

We expect that anxiety decreases at a faster rate in those in the pharmacogenetically- guided dosing arm compared to those randomized to standard dosing. Patients were included in the analyses regardless of whether they discontinued treatment, and missing data were accounted for with imputation modeling that accounts for dropout due to adverse events. Factors that may be incorporated in the mixed model include baseline characteristics (e.g., baseline anxiety severity and the type of primary anxiety disorder). Based on our randomized controlled trial of escitalopram for pediatric anxiety, we performed a power analysis using a mixed models procedure in PASS (100 simulations). This model allows us to account for the correlated nature of the longitudinal data. Based on our prior studies, we expected a 30.6% decrease in PARS score (equivalent to 6 units on a 25 point scale with 26 SD units) in patients receiving standard dosing [23,63]. Assuming a 2-unit difference (additional 10% reduction) in the overall mean PARS score (similar to the difference in IM and NMs receiving the same dose in [20]) between the two arms with 60 patients in each arm, we had 90% power at α = 0.05.

Activation was categorically analyzed, and the two arms were compared using logistic regression with factors differing by arm included as covariates. BMI changes over the 12-week treatment period were analyzed using mixed models to evaluate longitudinal changes. To evaluate the power to detect differences in weight gain, we used anthropomorphic data from a clinical trial, which evaluated change in weight in adolescents (same age as the sample for this study) who were treated with SSRIs [72], including the subset treated with es/citalopram to estimate effect size. Using the observed 3.94 standard deviation units for weight change when on es/citalopram, we had 80% power to detect a difference as small as 2.03 kg at α = 0.05 using 60 patients in each arm of the study. Given that the mean weight gain in children treated with es/citalopram without pharmacogenetically-guided dosing was 4.66 kg, this effect size is reasonable.

To evaluate the power to detect differences in discontinuation due to side effects, we considered both weight gain and activation. Prior studies have demonstrated a mean weight gain of 4.66 kg in children taking es/citalopram; thus, we expect approximately 50% of children in the standard arm to have a weight gain of ≥10 lbs (4.53 kg). Based on prior data, we expect 15% of children to experience activation while on es/citalopram. Assuming these two outcomes occur independently of each other, we expect 57.5% of patients who are randomized to standard dosing to have at least one of these outcomes. For the presence of either side effect, we have an 80% power to detect an odds ratio (OR) as small as 0.35 (equivalent to 32% in the pharmacogenetically-guided arm), with the side effect at α = 0.05 using 60 patients in each arm of the study. If we evaluate each side effect separately, we have an 80% power to detect an OR = 0.34 for weight gain and 0.07 for activation.

**Figure 3:**
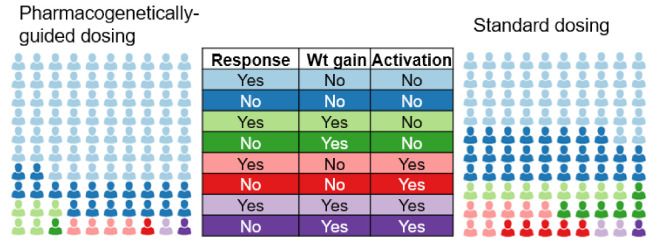
Expected response rates and rates of specific adverse events (weight gain and activation) in patients receiving pharmacogenetically-guided and standard escitalopram dosing.

We will also examine whether pharmacogenetically-guided dosing—compared to standard dosing—produces differences in secondary outcomes (e.g., response/remission by CGI score, discontinuation, Figure 3). For secondary outcomes, we will use a Cox Proportional Hazards analysis to evaluate the time to the event.

## 3. Impact of the Study

This study is unique in that it assesses the impact of CYP2C19-guided dosing on treatment outcomes in patients with anxiety disorders. Additionally, it extends findings from adult studies associating CYP2C19 metabolizer status with escitalopram exposure and investigates whether pharmacodynamic variants influence response, all of which have yet to be evaluated in a large pediatric population treated with escitalopram. Predicting the right dose for each patient has many benefits, including reductions in inpatient admissions, decreased side effect burden (and associated improvement in adherence), decreased cost of care, and reductions in cumulative morbidity and mortality (e.g., suicide attempts/completion that result from untreated or inadequately treated symptoms). We designed this study to address the significant gap in the evidence base for pharmacogenetically-guided treatment of pediatric patients with anxiety disorders that may also be applicable to patients with major depressive disorder.

## 4. Ethics Statement

The study was conducted according to the guidelines of the Declaration of Helsinki and approved by the Institutional Review Board of the University of Cincinnati (protocol number: 2020-0957, approved 9 October 2020). Additionally, a data safety monitoring board oversees the conduct of the study. ClinicalTrials.gov Identifier: NCT04623099.
